# Supplementary material for: High-throughput fitness screening and transcriptomics identify a role for a type IV secretion system in the pathogenesis of Crohn’s disease-associated Escherichia coli
Source: Nat Commun. 2021 Apr 1;12:2032. doi: 10.1038/s41467-021-22306-w (PMC8016931; doi:10.1038/s41467-021-22306-w)
Supplement: Supplementary file 1 — Supplementary Information [file 41467_2021_22306_MOESM1_ESM.pdf]

Supplementary Information

a

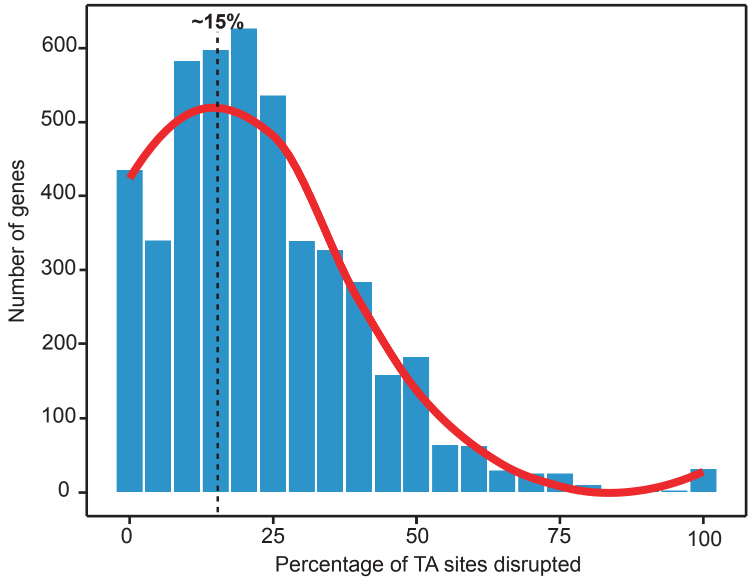

b

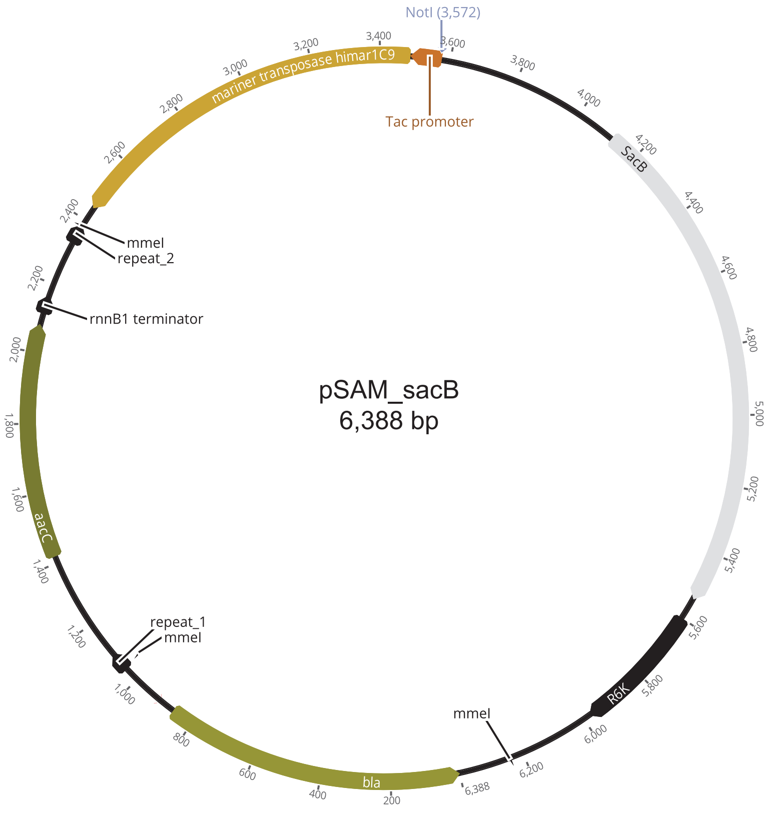

Supplementary Figure 1. Modifying the transposon mutagenesis system to increase AIEC library saturation. a. Histogram showing the percentage of “TA” sites disrupted per gene in AIEC strain NRG857c using the original transposon system. Shown is n = 1 of 3 technical replicates of the AIEC transposon mutant library that showed similar distribution. **b**. Map illustration of plasmid pSAM_SacB.

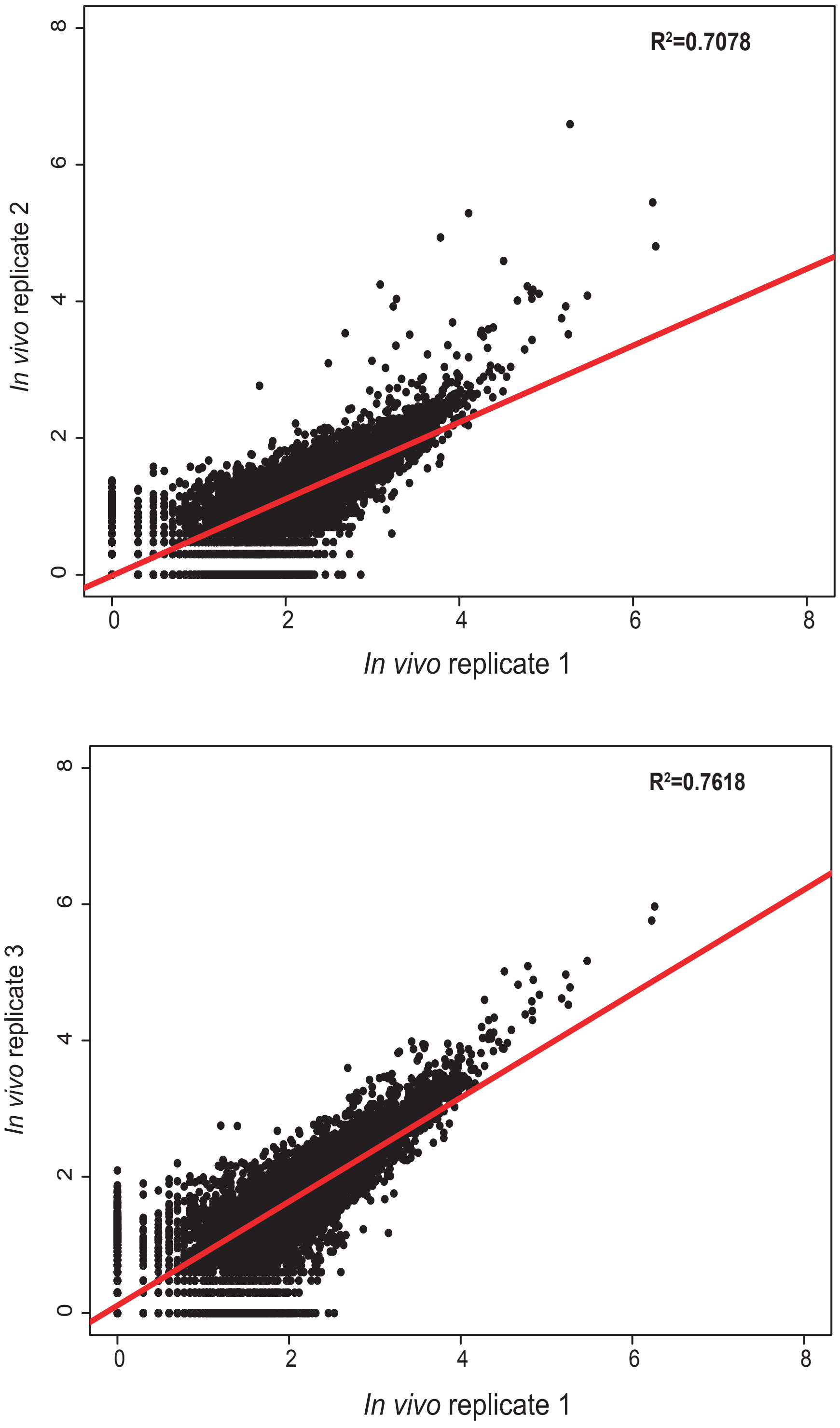

Supplementary Figure 2. **Correlation plots between different *in vivo* mutant libraries**. Each point represents the $\log_{10}$ value of the number of mapped reads of one mutant in each of two libraries. The correlation coefficient (R2) on log-transformed abundance values is shown.

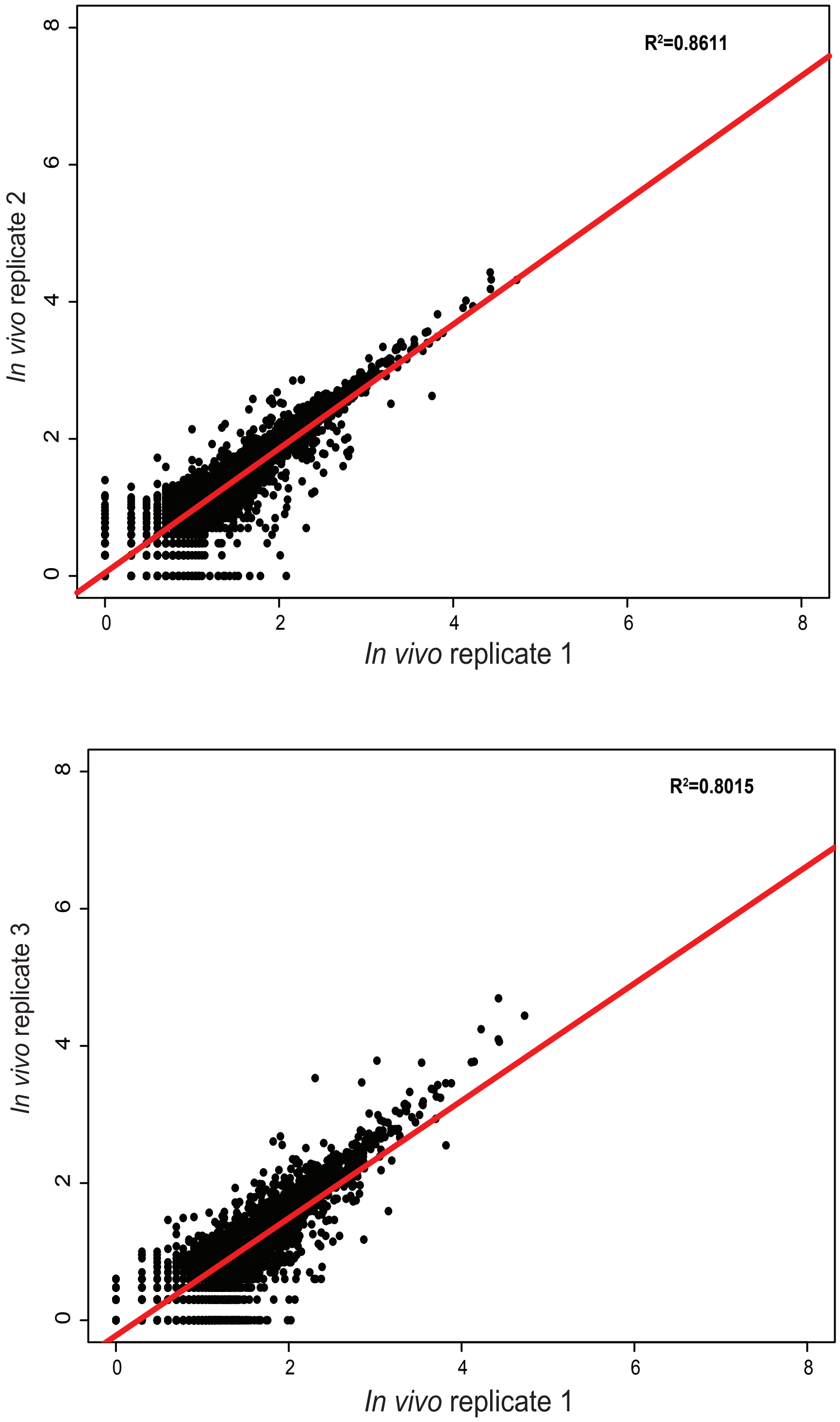

Supplementary Figure 3. Correlation plots between different *in vivo* transcriptomes. Each point represents the $\log_{10}$ value of the number of mapped reads per gene in each of the two libraries. The correlation coefficient (R2) on log-transformed abundance values is shown.
