# Supplementary material for: High-throughput fitness screening and transcriptomics identify a role for a type IV secretion system in the pathogenesis of Crohn’s disease-associated Escherichia coli
Source: Nat Commun. 2021 Apr 1;12:2032. doi: 10.1038/s41467-021-22306-w (PMC8016931; doi:10.1038/s41467-021-22306-w)
Supplement: Supplementary file 3 — Description of Additional Supplementary Files [file 41467_2021_22306_MOESM3_ESM.pdf]

Description of Additional Supplementary Files

File Name: Supplementary Data 1
Description: **Summary of the data from the *in vivo* genomic screens**. Shown for TIS are the fold change values of mutant abundance in the gut represented as change in read counts between different *in vivo* libraries and the inoculum. Shown for RNA-seq are Log_2 fold change values in gene expression of different AIEC loci in the host environment. COG assignment data of all genomic loci are reported as identified by eggNOG. Blank cells indicate that the loci could not be assigned to a particular COG family. The number of mapping reads and TA sites disrupted per gene are shown as separate sheets. Computation of statistical significance for TIS and RNA-sequencing data was constitutive of the analysis pipelines. For TIS, 100 Mann-Whitney U tests are performed for each locus to compare mutant abundance in each *in vivo* library to the corresponding 100 simulated control libraries. The average P value from the 100 Mann-Whitney U tests is reported for each genetic locus queried in each of the 3 biologically independent replicates. Loci showing less than 0.5-fold reduction in abundance at P values less than 0.01 in all 3 biological replicates were considered significant. For RNA-sequencing, DESeq2 performs a Wald test and reports P values that are adjusted for multiple testing using the procedure of Benjamini and Hochberg. Loci showing values >0.5 or <-0.5 for log_2 fold change in expression *in vivo* at adjusted P value < 0.05 were considered significant.

File Name: Supplementary Data 2
Description: **Primers used in the study**. Degenerate bases were included in the sequences of primers P7-Nx and P5-Nx to represent standard Illumina indices that were used in multiplexing different samples.
