# Supplementary material for: High-throughput fitness screening and transcriptomics identify a role for a type IV secretion system in the pathogenesis of Crohn’s disease-associated Escherichia coli
Source: Nat Commun. 2021 Apr 1;12:2032. doi: 10.1038/s41467-021-22306-w (PMC8016931; doi:10.1038/s41467-021-22306-w)
Supplement: Supplementary file 6 — Reporting Summary [file 41467_2021_22306_MOESM6_ESM.pdf]

nature research

Corresponding author(s): Coombes, Brian

Last updated by author(s): Feb 21, 2021

Reporting Summary

Nature Research wishes to improve the reproducibility of the work that we publish. This form provides structure for consistency and transparency in reporting. For further information on Nature Research policies, see our Editorial Policies and the Editorial Policy Checklist.

Statistics

For all statistical analyses, confirm that the following items are present in the figure legend, table legend, main text, or Methods section.

n/a	Confirmed	
☐	☒	The exact sample size (*n*) for each experimental group/condition, given as a discrete number and unit of measurement
☐	☒	A statement on whether measurements were taken from distinct samples or whether the same sample was measured repeatedly
☐	☒	The statistical test(s) used AND whether they are one- or two-sided<br>*Only common tests should be described solely by name; describe more complex techniques in the Methods section.*
☒	☐	A description of all covariates tested
☐	☒	A description of any assumptions or corrections, such as tests of normality and adjustment for multiple comparisons
☐	☒	A full description of the statistical parameters including central tendency (e.g. means) or other basic estimates (e.g. regression coefficient) AND variation (e.g. standard deviation) or associated estimates of uncertainty (e.g. confidence intervals)
☐	☒	For null hypothesis testing, the test statistic (e.g. *F*, *t*, *r*) with confidence intervals, effect sizes, degrees of freedom and *P* value noted<br>*Give P values as exact values whenever suitable.*
☒	☐	For Bayesian analysis, information on the choice of priors and Markov chain Monte Carlo settings
☒	☐	For hierarchical and complex designs, identification of the appropriate level for tests and full reporting of outcomes
☒	☐	Estimates of effect sizes (e.g. Cohen's *d*, Pearson's *r*), indicating how they were calculated

Our web collection on statistics for biologists contains articles on many of the points above.

Software and code

Policy information about availability of computer code

Data collection	Genomes of the E. coli strains shown in Figure 6a were retrieved from the NCBI and the repository of the Australian National University using the accession numbers provided in the original publications cited in the manuscript. MetaQuery was used to obtain the data shown in figure 6b. MetaQuery is a web application for rapid annotation and quantitative analysis of specific genes, functions, and taxa across >2,000 publicly available human gut metagenomes.
Data analysis	Data were analyzed using RStudio version 1.1.463 with R version 3.5.2, GraphPad Prism 9.0 software (GraphPad Inc., San Diego, CA), MATLAB version R2017b, previously published scripts by Pritchard JR et al (PLoS Genet., 2014), BWA version 0.7.17-r1188, cutadapt version 2.10, featureCounts version 1.6.4, bowtie2 version 2.3.5.1, EasyFig version 2.2.2, BLAST+ 2.10.0, eggNOG-mapper 4.5.1, ImageJ version 1.51,Pheatmap R Package version 1.0.10 and LightCycler software version 1.5

For manuscripts utilizing custom algorithms or software that are central to the research but not yet described in published literature, software must be made available to editors and reviewers. We strongly encourage code deposition in a community repository (e.g. GitHub). See the Nature Research guidelines for submitting code & software for further information.

Data

Policy information about availability of data

All manuscripts must include a data availability statement. This statement should provide the following information, where applicable:

- Accession codes, unique identifiers, or web links for publicly available datasets
- A list of figures that have associated raw data
- A description of any restrictions on data availability

Sequence data that support the findings of this study have been deposited in Sequence Read Archive (SRA) database and are accessible through the SRA accession number PRJNA684126 (https://www.ncbi.nlm.nih.gov/sra/?term=PRJNA684126). RNA-sequencing data that support the findings of this study have been deposited

in National Center for Biotechnology Information Gene Expression Omnibus (GEO) and are accessible through the GEO Series accession number GSM4969708 (https://www.ncbi.nlm.nih.gov/geo/query/acc.cgi?acc=GSM4969708). All other relevant data are available from the corresponding author upon reasonable request. Source data are provided with this paper.

Field-specific reporting

Please select the one below that is the best fit for your research. If you are not sure, read the appropriate sections before making your selection.

- [x] Life sciences
- [ ] Behavioural & social sciences
- [ ] Ecological, evolutionary & environmental sciences

For a reference copy of the document with all sections, see nature.com/documents/nr-reporting-summary-flat.pdf

Life sciences study design

All studies must disclose on these points even when the disclosure is negative.

Sample size	No assumptions regarding sample or effect size were made. Sample sizes were selected based on results from pilot experiments.
Data exclusions	No data were excluded from any analyses.
Replication	All experiments involved at least 3 biological replicates. All attempts at replication were successful.
Randomization	For animal experiments, mice were relocated at random from a housing cage to treatment or control cages. For other experiments, samples (for example in vitro-cultured cells) were randomly allocated to experimental groups.
Blinding	Microscopy examination was done blindly. For other experiments, the authors were not blinded due to the risk of confusion in handling samples. However, blinding was irrelevant in these experiments because all datapoints from experimental and control groups were used for statistical inference and drawing conclusions.

Reporting for specific materials, systems and methods

We require information from authors about some types of materials, experimental systems and methods used in many studies. Here, indicate whether each material, system or method listed is relevant to your study. If you are not sure if a list item applies to your research, read the appropriate section before selecting a response.

Materials & experimental systems

n/a	Involved in the study	
☐	☒	Antibodies
☐	☒	Eukaryotic cell lines
☒	☐	Palaeontology and archaeology
☐	☒	Animals and other organisms
☒	☐	Human research participants
☒	☐	Clinical data
☒	☐	Dual use research of concern

Methods

n/a	Involved in the study	
☒	☐	ChIP-seq
☒	☐	Flow cytometry
☒	☐	MRI-based neuroimaging

Antibodies

Antibodies used	E. coli O83 rabbit antiserum (SSI Diagnostica, Cat# 85077), 1:300 dilution<br>Goat anti-Rabbit IgG (H+L) Highly Cross-Adsorbed Secondary Antibody, Alexa Fluor 488 (Invitrogen, Cat# A-11034), 1:200 dilution.<br>Mouse anti-beta actin antibody (Abcam, Cat#ab8226), 1:500 dilution.<br>Goat anti-Mouse IgG (H+L) Highly Cross-Adsorbed Secondary Antibody, Alexa Fluor 568 (Invitrogen, Cat#A-11031), 1:200 dilution.
Validation	Primary antibodies were validated by the manufacturer as stated on their websites. For anti-O83, manufacturer (SSI Diagnostica) tested cross-reactivity with other O-antigens and reported none. For mouse anti-beta actin antibody, Abcam uses knockout cell lines to validate their antibodies.

Eukaryotic cell lines

Policy information about cell lines

Cell line source(s)	Caco-2 cell line were originally acquired from ATCC
Authentication	Morphology checks, growth curve analysis, species verification by isoenzymology, and other quality control tests were all performed on this cell line by the supplier at the time of distribution.

Mycoplasma contamination	Cells were regularly PCR-tested for mycoplasma contamination. All cell lines tested negative for mycoplasma contamination.
Commonly misidentified lines (See ICLAC register)	No commonly misidentified cell lines were used in the study.

Animals and other organisms

Policy information about studies involving animals; ARRIVE guidelines recommended for reporting animal research

Laboratory animals	Six-to-eight week old female C57BL/6 mice were purchased from Charles River Laboratories. Animals were housed in a specific pathogen free barrier unit under Level 2 conditions, temperature-controlled (21 °C), 30-50% humidity, 12h light and dark cycle environment (dark from 7pm to 7am) and were fed regular chow ad libitum.
Wild animals	No wild animals were used in the study.
Field-collected samples	No field collected samples were used in the study.
Ethics oversight	Animal experiments were conducted according to Canadian Council on Animal Care guidelines using protocols approved by the Animal Review Ethics Board at McMaster University under Animal Use Protocol #17-03-10.

Note that full information on the approval of the study protocol must also be provided in the manuscript.
